# New insights into the raw milk microbiota diversity from animals with a different genetic predisposition for feed efficiency and resilience to mastitis

**DOI:** 10.1038/s41598-022-17418-2

**Published:** 2022-08-05

**Authors:** Armin Tarrah, Simone Callegaro, Shadi Pakroo, Raffaella Finocchiaro, Alessio Giacomini, Viviana Corich, Martino Cassandro

**Affiliations:** 1 Department of Agronomy, Food, Natural resources, Animals and Environment (DAFNAE), University of Padova, Viale dell’Università 16, 35020 Legnaro, PD Italy; 2 Associazione Nazionale Allevatori Razza Frisona, Bruna e Jersey Italiana—ANAFIBJ, 26100 Cremona, Italy; 3 Associazione Nazionale Allevatori Delle Razze Bovine Charolaise E Limousine Italiane (ANACLI), 00187 Roma, Italy; 4 Present Address: Department of Food Science, Canadian Research Institute for Food Safety, University of Guelph, Guelph, ON N1G 2W1 Canada

**Keywords:** Microbiology, Zoology

## Abstract

The main objective of this study was to assess the microbiota diversity in milk samples collected from Holstein cows with different estimated breeding values for predicted feed efficiency, milk coagulation, resilience to mastitis, and consequently, to study its effects on milk quality. One hundred and twenty milk samples were collected in two seasons (summer and winter) from different commercial dairy farms in the Nord-east of Italy. For each trait, 20 animals divided into two groups of the high (10 cows) and the low (10 cows) were selected to study the microbiota profile using 16S rRNA metabarcoding sequencing. The alpha and beta diversity analysis revealed significant differences between the high and the low groups for feed efficiency and resilience to mastitis, while no significant difference was detected for milk coagulation. Moreover, remarkable differences among the taxa were detected between the two seasons, where the winter was more diverse than summer when applied the Chao1 index. Lastly, the linear discriminant analysis (LDA) effect size (LEfSe) indicated *Aerococcus, Corynebacterium, Facklamia,* and *Psychrobacter* taxa with more abundance in the high group of feed efficiency, whereas, in resilience to mastitis, only two genera of *Mycoplana* and *Rhodococcus* were more abundant in the low group. In addition, LEfSe analysis between the seasons showed significant differences in the abundance of *Bacteroides*, *Lactobacillus*, *Corynebacterium*, *Escherichia*, *Citrobacter*, *Pantoea*, *Pseudomonas*, and *Stenotrophomonas*. These findings indicate that the different genetic predisposition for feed efficiency and resilience to mastitis could affect the raw milk microbiota and, consequently, its quality. Moreover, we found more abundance of mastitis-associated bacteria in the milk of dairy cows with a higher feed efficiency index.

## Introduction

Milk contains different types of microorganisms. The most predominant microorganisms that compose milk microbiota include various species of *Lactococcus*, *Lactobacillus, Pseudomonas, Micrococcus, Staphylococcus*, and yeast^1,2^. Other microbial groups in raw milk belong to the *Leuconostoc, Enterococcus*, *Streptococcus*, *Bacillus*, *Clostridium*, *Listeria*, and *Enterobacteriaceae.* Besides, many gram-negative bacteria such as *Acinetobacter, Alcaligenes, Flavobacterium*, and *Aeromonas* species^1^. Milk is a vibrant food with high nutritional value since it contains all essential food ingredients like minerals, protein, fat, and lactose^3^. Based on recent studies, milk microbiota composition can contribute to milk quality to a great extent; notably, some bacteria, such as lactic acid bacteria, can play a critical role in high milk quality^4,5^.

Milk can be examined with both culture-dependent and culture-independent approaches^6,7^. During the last years, molecular techniques such as 16S rRNA metabarcoding have become more popular since using the 16S rRNA sequencing method allows us to study both live and dead bacteria while we lose the dead cells by culture-dependent technique in that environment^8^.

Nowadays, dairy consumers pay more attention to the safety and sensory quality of milk and dairy products compared to the past, which could depend on different factors such as cattle's diet and breed, milk processing (e.g., pasteurization), and milk preparation^9^.

Feed efficiency is one of the most critical factors in milk production, which means, evaluation of the cow's ability to convert the amount of food that has been received to the amount of milk that has been produced (the ratio of milk yield to dry matter intake^10^). Feed efficiency improvement has resulted from high milk yield-oriented breeding programs and proper feeding and management of animals over the last years; however, its effect on milk microbiota and microbial milk quality has not been appropriately studied yet^10^.

Bovine mastitis is an inflammatory reaction of the udder tissue usually caused by various microorganisms, which is a mammary gland infection with a high rate of prevalence in dairy cattle worldwide^11,12^. Mastitis disease is another factor that can reduce the quality and quantity of the milk coming from infected cows and needs particular attention due to its possible health and economic damages^11,13,14^.

Milk coagulation properties are another trait economically relevant for the dairy industry due to its effect on cheese quality, quantity, and cheese-making efficiency^15^. Many factors such as production season, processing company, microbiota, breed, stage of lactation, parity, and udder health status can affect the milk coagulation thickness and time^16^.

Overall more than 70% of milk production is used to manufacture cheese in Italy, and 55% of the total milk production is processed for PDO (Protected Designation of Origin) cheeses like Grana Padano, Provolone, Gorgonzola, Parmigiano-Reggiano, Asiago, and Mozzarella^17^. The main objective of the current research was to investigate the raw milk microbiota diversity in milk samples collected from cows with different estimated breeding values (EBVs) for feed efficiency, milk coagulation traits, resilience to mastitis, and consequently, to study its effects on milk bacterial community.

## Results and discussion

In the current study, the microbial community of 120 raw milk samples collected in two different seasons (60 samples during summer and 60 samples during winter) from 60 Italian Holstein Friesian cows based on different EBVs for FE, RM, and MC, has been investigated. The average EBV for the high FE group was calculated at 1.48 ± 0.05 (kg of milk yielded/ kg of dry matter intake), while the low group was 1.19 ± 0.12 (kg of milk yielded/ kg of dry matter intake). Regarding the RM, the average EBV for the high group was determined at 111.41 ± 1.92, whereas the low group was 88.08 ± 2.42. Lastly, the average BLUP value for the high MC group was calculated at 114.74 ± 0.099, while for the low group was 85.61 ± 0.63. Animal selection for traits such as feed efficiency or resilience to mastitis may potentially affect final raw milk microbiota composition and its final quality. In several studies, strong positive genetic and phenotypic correlations between milk yield and feed efficiency have been reported^18,19^. Derakhshani et al.^20^also observed that udder inflammation related to mastitis was negatively correlated with milk microbiota; therefore, resilience to mastitis could affect milk microbiota composition as well. Hence, animal selection based on different genetic predispositions for feed efficiency and resilience to mastitis could be a possible indicator of final raw milk microbiota composition.

A total of 3,441,325 high-quality reads with an average read count of 28,677 per sample were obtained after removing low-quality and chimeric sequences using a 16S rRNA metabarcoding analysis of the V4 hypervariable region and MiSeq sequencing system. After clustering the OUT table based on classification at a 97% similarity using BLASTn, 407 OTUs were considered for analysis after removing low abundance and low variance features as explained in the “Material and methods” section. Overall, Proteobacteria and Firmicutes, both with 32%, were the most predominant at the phylum level, followed by Actinobacteria (29%) and Bacteroidetes (6%). The rarefaction curves (Supplementary Fig. 1) and the Good's coverage index average of 99.51 ± 0.42 showed that the sequencing depth has been sufficient to cover the bacterial species richness of all analyzed raw milk samples in this study.

**Figure 1:**
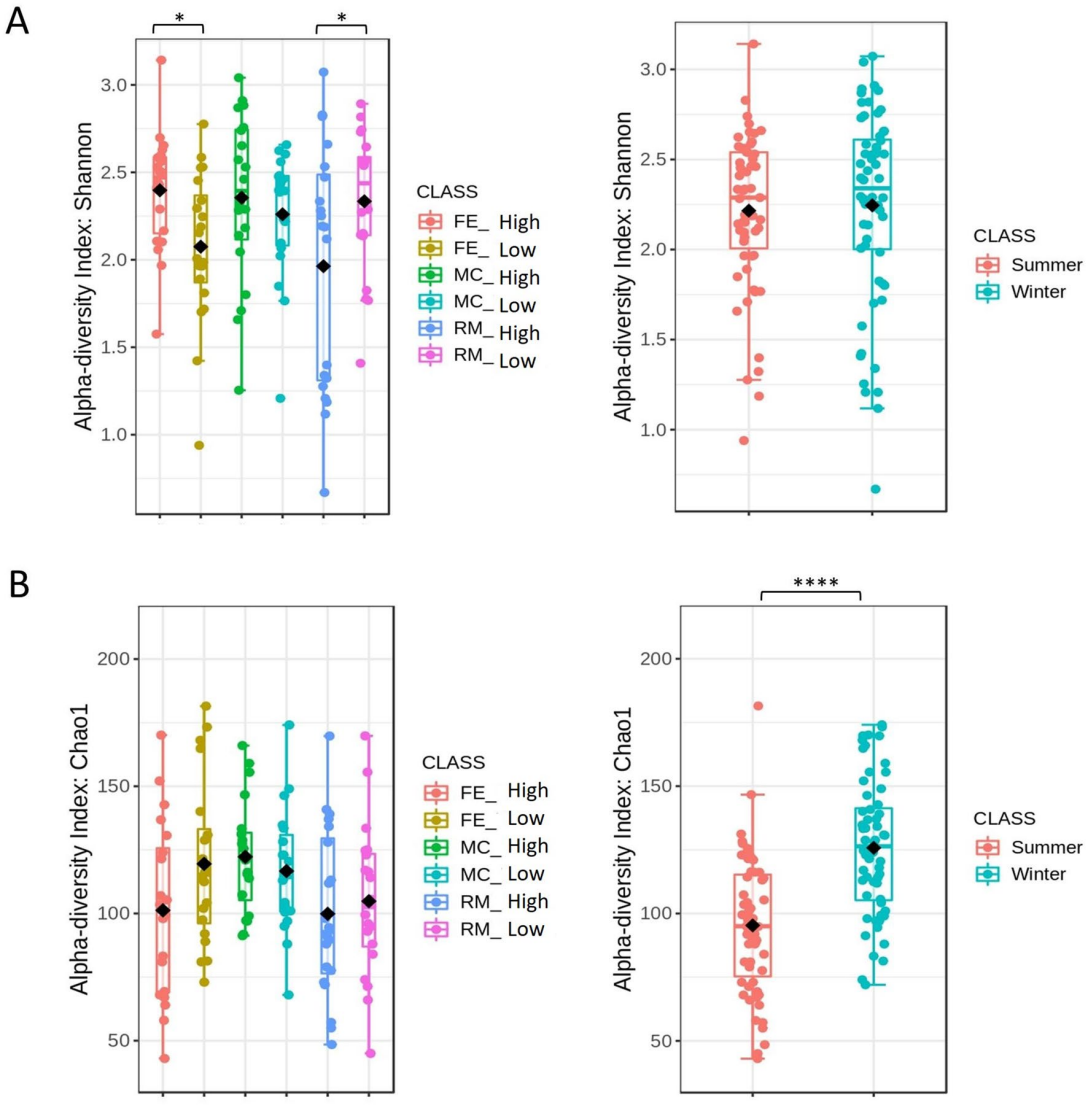
Alpha diversity using the Shannon (**A**) and Chao1 (**B**) indexes. Asterisks represent statistically significant differences between the high and low groups within each trait/season (*p ≤ 0.05; ** *p* ≤ 0.01; *** *p* ≤ 0.001; **** *p* ≤ 0.0001).

The Shannon index, accounting for species evenness, revealed significant differences in the alpha diversity between the high and the low groups of FE and RM (*p* < 0.05); however, no significant differences was detected in MC (high and low) as well as between summer and winter (Fig. 1). Regarding the FE, we have seen higher diversity in the high group, while, in RM, the situation was contrary (Fig. 1). Moreover, the Chao1 index, accounting for the species richness, showed a significant difference (*p* < 0.0001) only between the seasons, where the winter appeared as a more diverse season (Fig. 1).

**Figure 2:**
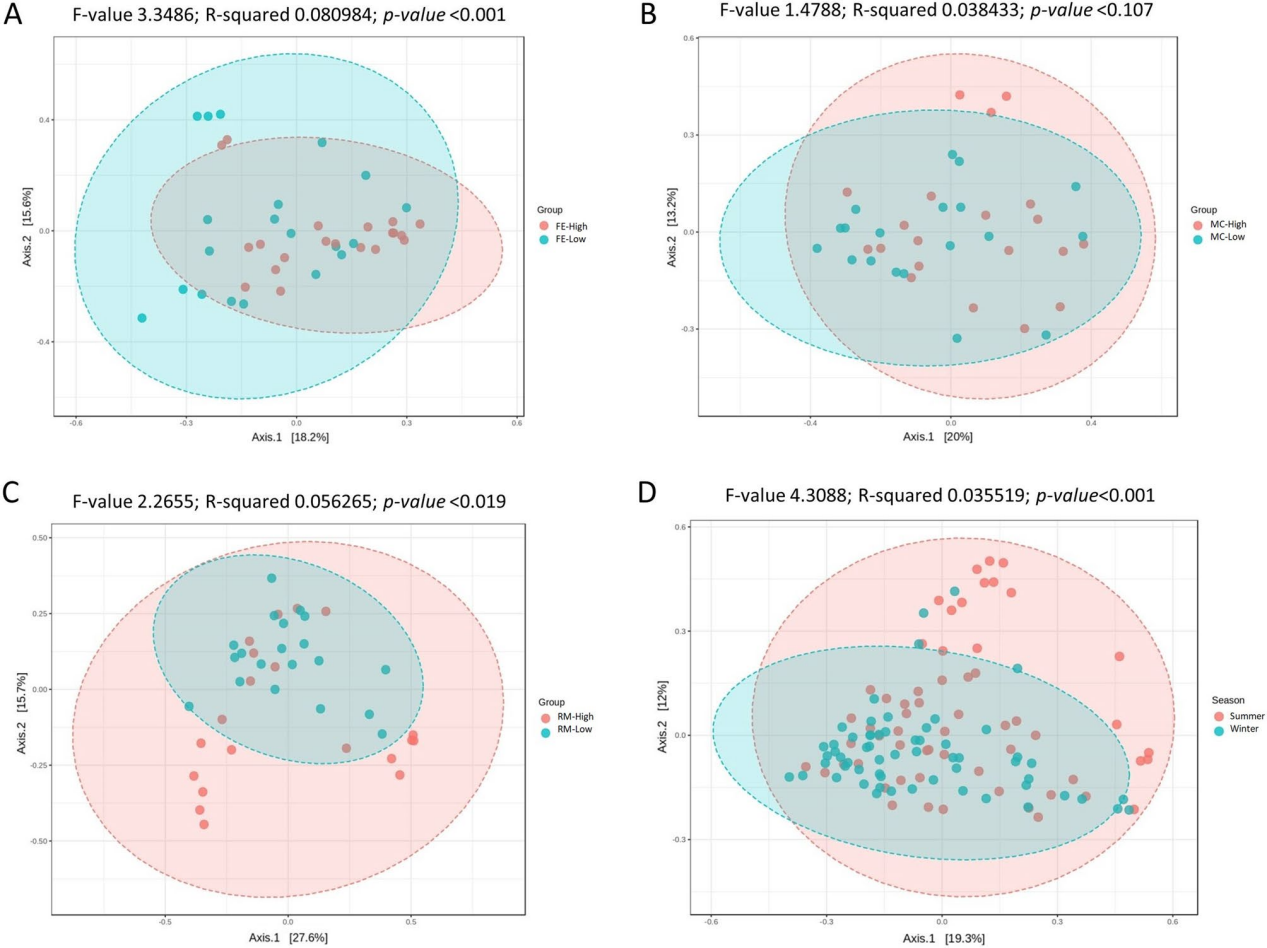
Principal coordinate analysis (PCoA) based on Bray–Curtis distance metrics and permutational multivariate analysis of variance (PERMANOVA) to indicate the beta diversity among different groups. (**A**) Feed Efficiency, (**B**) Milk Coagulation, (**C**) Resilience to Mastitis, (**D**) Different seasons.

The diversity analysis using the Bray–Curtis index and PERMANOVA as statistical method indicated significant differences in microbial structure between the groups (high and low) of FE (F-value 3.3486; R^2^ 0.080984; *p* value < 0.001) and RM (F-value 2.2655; R^2^ 0.056265; *p* value < 0.019); however, no significant difference (F-value 1.4788; R^2^ 0.038433; *p* value < 0.107) was recorded between the groups of MC (Fig. 2). The same analysis between the two seasons also showed significant differences (F-value 4.3088; R^2^ 0.035519; *p* value < 0.001), indicating a high beta diversity in microbial structure when two seasons were compared with each other (Fig. 2).

**Figure 3:**
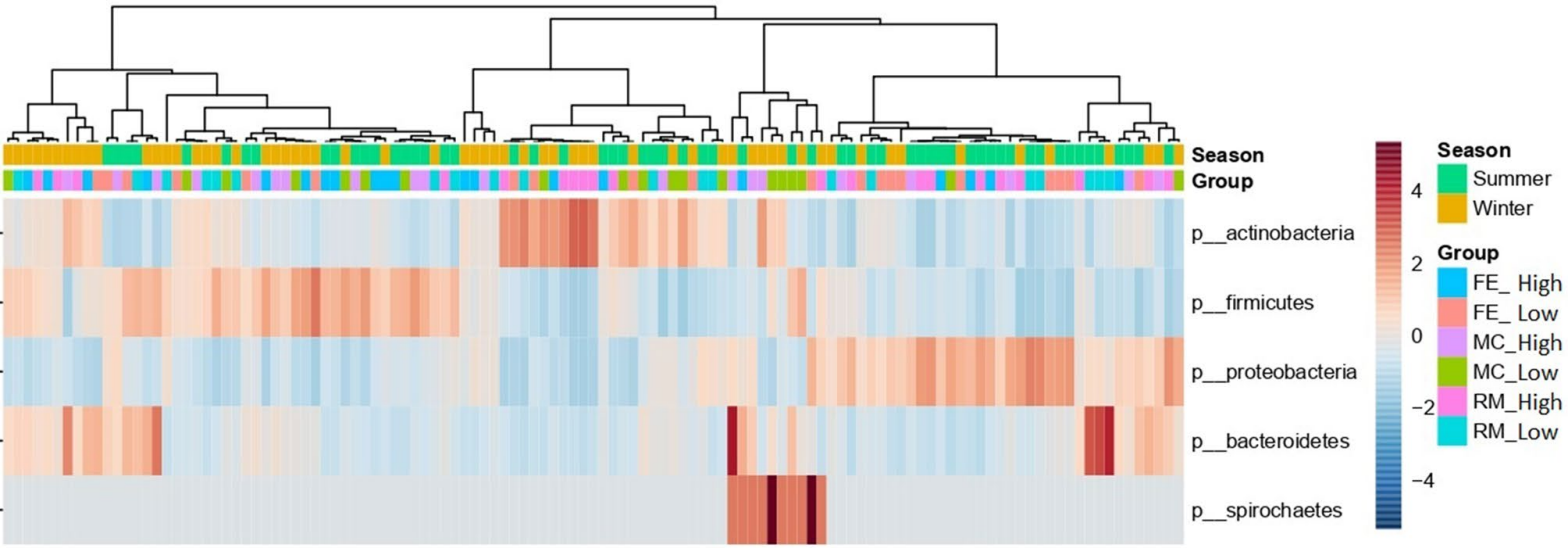
Heat map based on the relative abundance of bacteria among the groups and seasons at the phylum level using the linear discriminant analysis (LDA) effect size (LEfSe).

Finally, the LEfSe analysis unfolded the differences between OTUs among the groups. At the phylum level, the high and low groups related to MC and RM indicated similar composition; however, in FE, the high group showed Actinobacteria with 35% as the predominant phylum, while, in the low group, Firmicutes with 40% was the most abundant phylum, and it was significantly more abundant compared to the high group (LDA = 5.8) (Fig. 3). Regarding different seasons, we have seen quite different microbial compositions between the two seasons. In winter, Actinobacteria (35%), Firmicutes (34%), and Proteobacteria (24%) were predominant phyla respectively, while, in the summer, Proteobacteria (41%) Firmicutes (30%) and Actinobacteria (24%) were the most abundant phyla respectively (Fig. 3). It is worth mentioning that Proteobacteria and Actinobacteria were significantly different, with the LDA of 5.9 and 5.7 between the two seasons, which shows a great effect size (Fig. 3).

**Figure 4:**
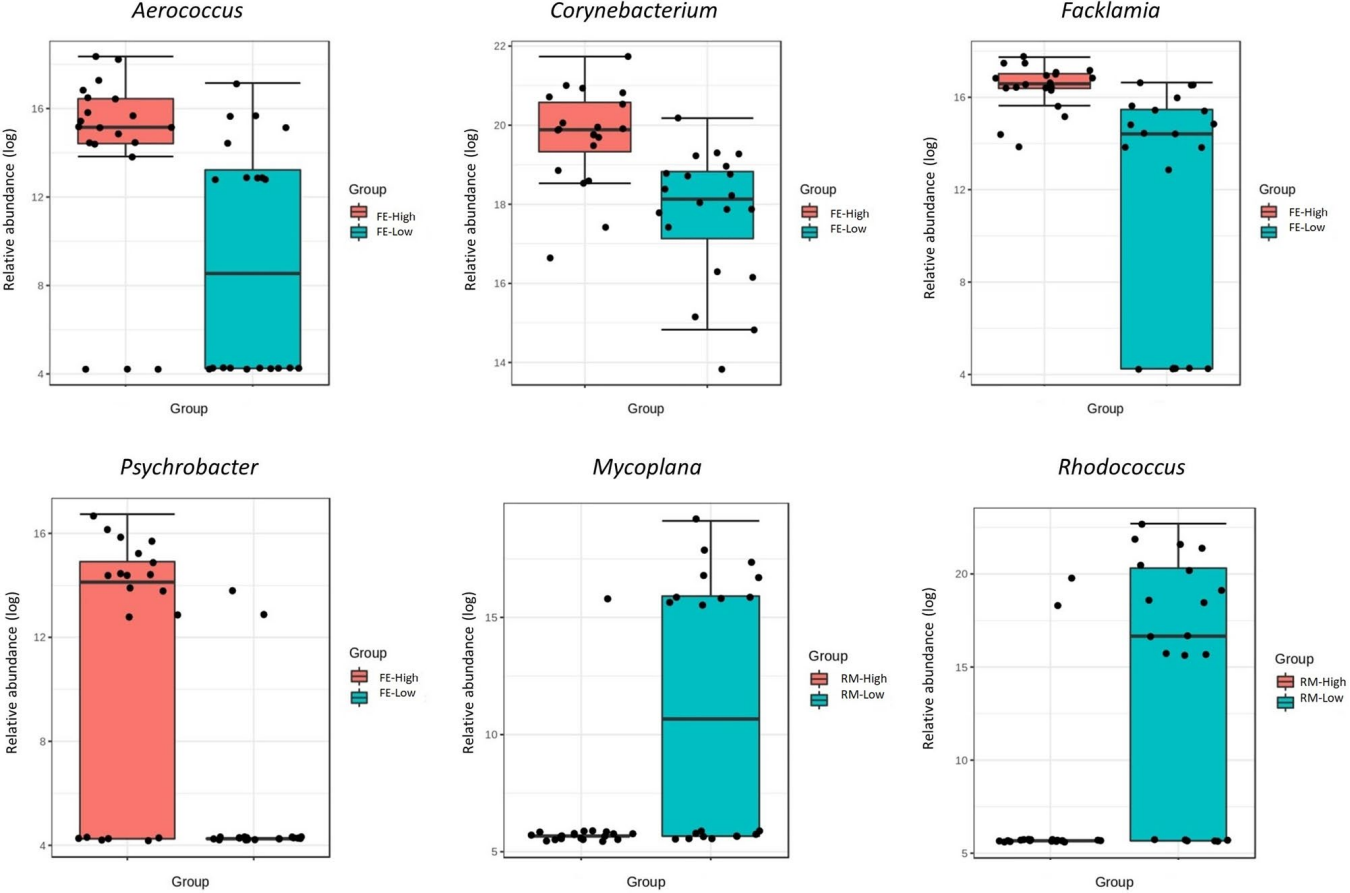
Linear discriminant analysis Effect Size (LEfSe) at genus level between the high and low groups within each trait. An LDA score greater than 2 was used to determine significantly different genera between the groups.

Using the same analysis (LEfSe) at the genus level revealed significant differences between FE and RM's high and low groups. Regarding the FE, *Aerococcus, Corynebacterium, Facklamia,* and *Psychrobacter* were significantly (LDA > 2) more abundant in the high group (Fig. 4). On the other side, in RM, two genera were significantly different (LDA > 2); *Mycoplana* and *Rhodococcus,* which were both more abundant in the low group (Fig. 4).

**Figure 5:**
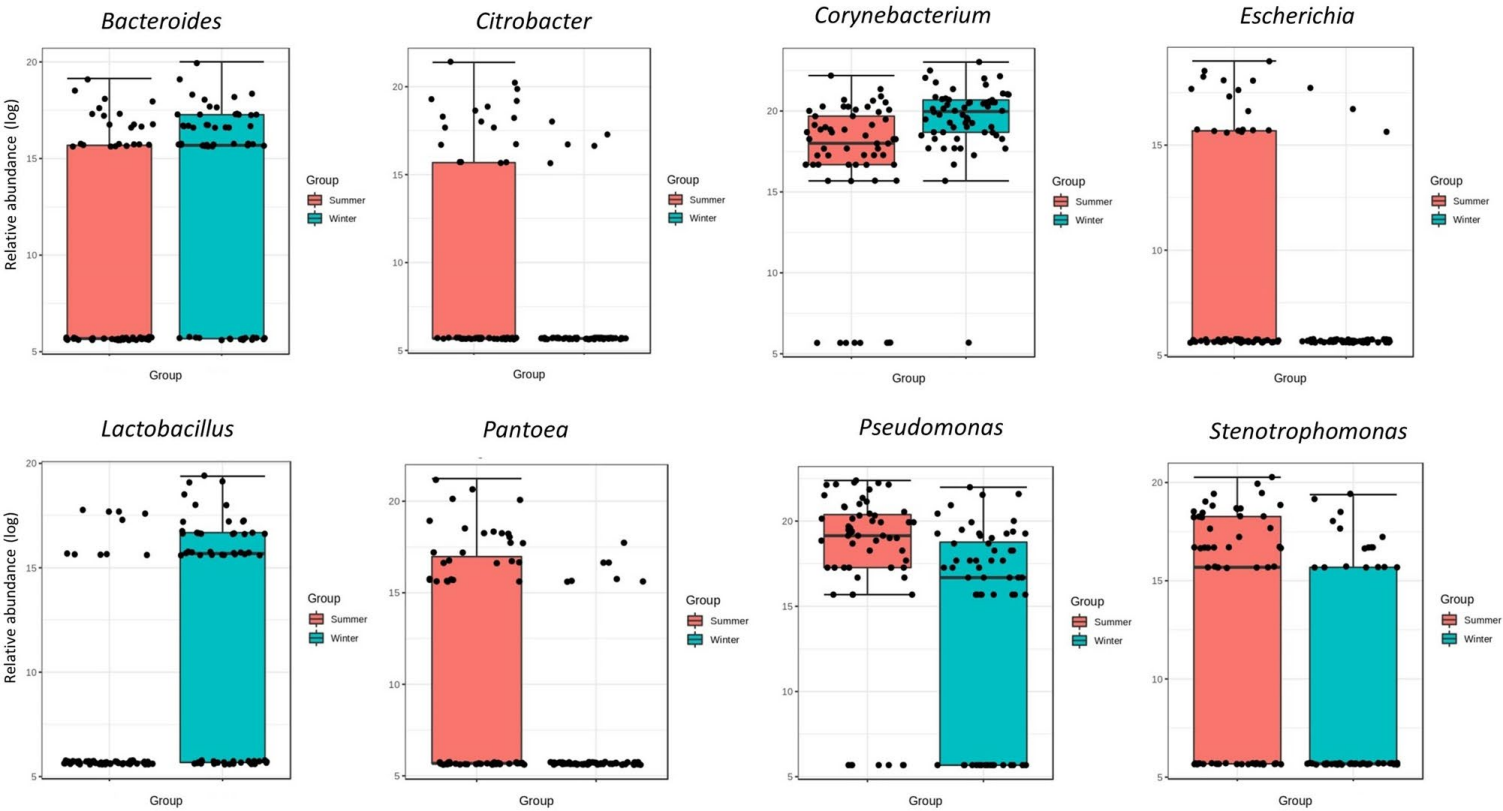
Linear discriminant analysis Effect Size (LEfSe) at genus level between the summer and winter. An LDA score greater than 2 was used to determine significantly different genera between the seasons.

In addition, LEfSe analysis between the seasons showed significant differences in eight genera; *Bacteroides*, *Lactobacillus*, *Corynebacterium*, *Escherichia*, *Citrobacter*, *Pantoea*, *Pseudomonas*, and *Stenotrophomonas* (Fig. 5). The genera *Bacteroides*, *Lactobacillus*, and *Corynebacterium* were relatively more abundant in winter, while *Escherichia*, *Citrobacter*, *Pantoea*, *Pseudomonas*, and *Stenotrophomonas* were less abundant in comparison with summer (Fig. 5).

Based on recent studies, the composition of milk microbiota can affect milk quality to a great extent; remarkably, some microbes, such as lactic acid bacteria, can play a vital role in high milk quality^4,5^. On the other side, spoilage and pathogenic bacteria can lead to low-quality milk, health threat, and, consequently, economic damage for the dairy industry. Interestingly, we have detected significant differences between FE and RM's high and low groups. Among the *Aerococcus* species, *A. viridans* is the only species related to the dairy environment, and it has been isolated from clinical and subclinical intramammary infections in dairy cows^21^. In a study by Spakova et al*.*^22^, 12 *A. viridans* strains were isolated from clinical and subclinical cases in Slovakia, indicating its relevance in bovine mastitis. Together with *Streptococcus*, *Escherichia*, *Staphylococcus*, and *Enterococcus*, members of the *Corynebacterium* genus represent the most relevant mastitis-related bacteria in dairy farming^23^. Among the corynebacteria, *Corynebacterium bovis* is the most isolated bacterium from bovine subclinical mastitis, followed by *C. minutissimum*, *C. ulcerans*, *C. amycolatum*, and *C. pseudotuberculosis,* which are all associated with clinical or subclinical bovine mastitis as well^24,25^. *Facklamia* is a Gram-positive genus of bacteria from the family of *Aerococcaceae*, which has members with pathogenic characteristics^26^. In several recent studies, the abundance of *Aerococcus*, *Corynebacterium*, and *Facklamia* was significantly higher in cows with a clinical mastitis history than in healthy cows^4,27^.

In some studies, it has been reported the increase of bacterial richness in the summer season compared to winter^28,29^; our finding did not indicate any significant differences for bacterial evenness between the two seasons; however, we had a significantly (*p* < 0.0001) greater bacterial richness in winter which is in contrary with abovementioned studies. Indeed, another study by Olde Riekerink et al*.* reported that greater bacterial richness during winter could happen due to the appearance of psychrophilic milk spoilage and mastitis-related bacteria such as *S. aureus* and *S. dysgalactiae*^30^. In our study, *Bacteroides*, *Lactobacillus*, and *Corynebacterium* were more abundant in winter, whereas the *Escherichia*, *Citrobacter*, *Pantoea*, *Pseudomonas*, and *Stenotrophomonas* indicated the greater abundance in summer. These seasonal differences in milk microbiota might be related to milk exposure time to the environment, resulting in a greater diversity of bacteria in winter^31^. Other possibilities could also be related to the differences in housing and feeding during summer and winter^31^.

This study characterized the bovines' raw milk bacterial community with different estimated breeding values for traits such as feed efficiency, milk coagulation, and resilience to mastitis. Although no significant differences were detected when the groups of milk coagulation were compared at different taxonomy levels with each other; however, regarding the feed efficiency, the outcome of microbiota diversity analysis revealed the increase of genera *Aerococcus*, *Corynebacterium*, *Facklamia* that are all related to prevalence of dairy cow mastitis. Based on collected results in this research, the dairy cows with the higher feed efficiency indicated more abundance of mastitis-associated bacterial taxa in their raw milk microbiota. On the other side, the low group of resilience to mastitis showed more abundance of *Rhodococcus* among the microbiota. This bacterium has also been reported frequently as a mastitis-associated microorganism. The current study's findings indicate that the different genetic predisposition for feed efficiency and resilience to mastitis could affect the raw milk microbiota diversity.

## Material and methods

### Animal selection

Sixty Italian Holstein Friesian cows from 28 commercial dairy farms, all located in Veneto Region, Italy, were selected based on their estimated breeding values for: (1) predicted feed efficiency (FE), (2) resilience to mastitis (RM), and (3) milk coagulation trait (MC). For each trait (FE, MC, or RM), 20 animals (10 cows with the high and 10 cows with the low estimated breeding values) were selected to be studied and compared regarding the raw milk bacterial diversity in two different seasons (summer and winter 2020).

All animals rearing and handling procedures were carried out in accordance with the European Commission recommendation 2007/526/EC and Directive 2010/63/UE on revised guidelines for the accommodation and care of animals used for experimentation and other scientific purposes.

Regarding resilience to mastitis, the index was created using four novel traits that have shown the strongest genetic correlation. These traits are the mean of somatic cell count (SCC) between 5 and 150 days in milk (DIM), the standard deviation of SCC within lactation, severity, defined as the ratio between the number of test-days with SCC greater than 400,000 cells/mL and the total number of test-days within lactation and peak defined as the number of peaks during lactation (number of times when SCC shows a change from < 100,000 to 400,000 cells/ml on three consecutive test-days). The environmental effects considered in the model were herd-year-season, age at first calving, and the number of test-day records within lactation. The random effect was the animal effect, considering bulls and cows simultaneously, accounting for the genetic level of mating and using all available pedigree information. Heritability (h2) of the aggregate udder health index was 15%. The index was expressed on a scale with a mean of 100 and SD of 5. The genomic index was calculated following the following steps: genotyping individual animals, collecting phenotypes and estimating the value of the individual markers (SNP). The model used to estimate these effects includes the 68,000 markers obtained after the initial editing procedures and a so-called “polygenic” effect, which was included in the model through the kinship information of the animals. The part of the variability not explained by the genetic markers can be recovered by incorporating kinships between the animals into the model:

$$ {\text{DGV}} = {\text{Markers}} + {\text{Classic kinship}} + {\text{error}} $$

For bulls, a direct genomic index can be calculated both for sires with daughters and for bulls without daughters (young bulls) after the value of the individual markers has been determined. Direct genomic information was combined with the traditional index for sires with daughters to increase reliability. The same genomic approach used for males was also applied to cows.

For milk coagulation properties, the main milk coagulation traits studied are milk rennet coagulation time (RCT, min) and curd firmness (a30, mm), but these two individual traits have been combined according to Penasa et al*.*^32^ to give the aggregate index of milk aptitude to coagulate. Milk coagulation was introduced as a new standardized trait to summarise RCT and a30, with the same index importance (50%). The following linear model was used to predict EBV of milk coagulation trait:

$$ {\text{yijklm}} = {\text{mean}} + {\text{Herd-Year-Seasoni}} + {\text{DIMj}} + {\text{Parityk}} + {\text{animl}} + \upvarepsilon {\text{ijklm}} $$

where yijklm is a measure of the milk coagulation trait; mean is the general mean of the model; Herd-Year-Seasoni (i = 1, …, n) is the fixed effect of herd-year-season; DIMj (j = 1, …, 14) is the fixed effect of DIM; Parityk (k = 1, 2, 3) is the fixed effect of parity; animl is the random additive genetic effect of an animal l, N(0, Aσ2a); εijklm is a random residual effect, N(0, Iσ2ε). Animal and residual effects were assumed to be independent. The pedigree information consisted of at least 3 generations for each cow with a record. In the model, the herd and test-day effects were con-founded because cows in each herd were sampled only once, all on the same test day. Days in milk of each cow were grouped into 10 monthly classes from 5 to 305 d after calving, 3 bimonthly classes from 306 to 486 d, and 1 class for records collected after 486 d. Parity was classified into 3 classes for first, second and third to seventh calving as well.

Finally, predicted feed efficiency recording data are available in the ANAFIBJ database, coming from milk recordings and type evaluations. Starting from this information and the age of the animal, the bodyweight (BW) was estimated based on the predicted BW and the fat corrected milk production (FCM). An estimation of the dry matter intake (DMI) was made. Predicted feed efficiency was calculated as the ratio between the energy corrected milk production (ECM, which considers both the fat and protein percentages) and the predicted DMI. The model was an animal model with repeated measures. The environmental effects (fixed effects) taken into account are the interaction between calving age and parity, the interaction between days in milk (gathered into classes of 30 days each) and parity and the herd-year-recording day effect. The animal and the permanent environmental effect were considered as random effects. The predicted feed efficiency index was expressed on scale 100 and SD of 5 just like for the other functional traits. The bulls with index greater than 100, transmit a higher feed efficiency than the average of the genetic base.

### Milk sample collection

One hundred and twenty raw milk samples were collected during the summer (60 samples) and winter (60 samples) in 2020. It is worth mentioning that the same animals were used for both seasons. The milk samples were collected through an automatic sampler installed in the milking parlor during the evening and after cleaning bovine teats with osmosis water and 70% ethanol.

After the sample collection, 200 µL of Bronopol (2-bromo-2-nitropropan-1,3-diol) was used in 40 mL of milk as the preservative, and the milk samples were transferred at 4 °C to the food microbiology laboratory of the University of Padova (DAFNAE, Padova, Italy).

### 16S rRNA metabarcoding sequencing

All 120 raw milk samples collected during summer and winter were thawed on ice, 10 mL milk was centrifuged at 5500*g* for 20 min at 4 °C and the pellet were used for gDNA extraction using the DNeasy PowerSoil Kit (Qiagen, Hilden, Germany) according to the manufacturer instructions. Genomic DNA was sent for Next-generation sequencing (NGS) at Molecular Research DNA (MRDNA, Shallowater, TX, USA). The hypervariable region V4 of the the16S rRNA gene was chosen to study the microbial profile. The universal primers 515/806 were used to amplify the V4 region in a 30-cycle PCR using the HotStarTaq Plus Master Mix Kit (Qiagen, USA) using the following conditions: 95 °C for 5 min, followed by 30–35 cycles of 95 °C for 30 s, 53 °C for 40 s and 72 °C for 1 min, after which a final elongation step at 72 °C for 10 min was performed. Later, 2% agarose gel was used to assess the amplification success and intensity of the bands. Multiple samples were pooled together in equal proportions based on their DNA concentrations and molecular weight, purified using calibrated Ampure XP beads, and the Illumina DNA library was prepared. Sequencing was performed on a MiSeq (MR DNA) following the manufacturer's guidelines, generating 250 bp paired-end (PE) reads.

### Sequence read processing and analysis

Sequence data were processed using the Molecular Research DNA analysis pipeline (MR DNA). Briefly, sequences were joined, barcodes, sequences < 150 bp, and ambiguous base calls were removed. Then, sequences were denoised, operational taxonomic units (OTUs) generated, and chimeras depleted. Final OTUs were taxonomically classified at a 97% similarity using BLASTn. Raw reads were deposited in the Sequence Read Archive (SRA) database under the BioProject number “PRJNA789190”.

### Methods of comparison and statistical analysis

The statistical analysis was carried out using the MicrobiomeAnalyst server^33^. The OTU table and the experimental design metadata file were imported into the MicrobiomeAnalyst server, where the low count/variance sequences removal and total sum scaling (TSS) conduction was performed using default parameters, and the OTU tables were rarefied to the minimum library of 12,837 sequences to avoid bias due to sequencing depth among different samples.

The alpha diversity was calculated using Shannon and Chao1 indexes^34,35^. In each trait (FE, RM, and MC), the high and the low groups were compared by setting the statistical approach on t-test/ANOVA. The same approach was used to compare the alpha diversity between the summer and winter seasons.

Moreover, the principal coordinate analysis (PCoA), the Bray–Curtis index, and permutational multivariate analysis of variance (PERMANOVA) were chosen as orientation, distance, and statistical methods to calculate the beta diversity among the groups.

Finally, the significant OTUs differences between the high and the low groups in each trait (FE, RM, and MC) were analyzed using the linear discriminant analysis (LDA) effect size (LEfSe) tool using default parameters. The same approach was used to compare the significant OTUs differences between the summer and winter seasons.

## Supplementary Information

Supplementary Information 1.

Supplementary Information 2.

## Data Availability

The raw reads were deposited publicly in the Sequence Read Archive (SRA) database under the BioProject number “PRJNA789190”.
