# Supplementary material for: New insights into the raw milk microbiota diversity from animals with a different genetic predisposition for feed efficiency and resilience to mastitis
Source: Sci Rep. 2022 Aug 5;12:13498. doi: 10.1038/s41598-022-17418-2 (PMC9356063; doi:10.1038/s41598-022-17418-2)
Supplement: Supplementary file 1 — Supplementary Information 1. [file 41598_2022_17418_MOESM1_ESM.docx]

**New insights into the raw milk microbiota diversity from animals with a different genetic predisposition for feed efficiency and resilience to mastitis**

Armin Tarrah^1†^, Simone Callegaro^1,3^, Shadi Pakroo^1^, Raffaella Finocchiaro^2^, Alessio Giacomini^1^, Viviana Corich^1*^, Martino Cassandro^1,2^

^1^ Department of Agronomy, Food, Natural resources, Animals and Environment (DAFNAE), University of Padova, Viale dell’Università 16, 35020 Legnaro (PD), Italy

^2^Associazione Nazionale Allevatori Razza Frisona, Bruna e Jersey Italiana—ANAFIBJ, 26100 Cremona, Italy

^3^Associazione Nazionale Allevatori delle Razze Bovine Charolaise e Limousine Italiane (ANACLI), 00187 Roma, Italy

^†^Current address: Department of Food Science, Canadian Research Institute for Food Safety, University of Guelph, Guelph, ON N1G 2W1, Canada


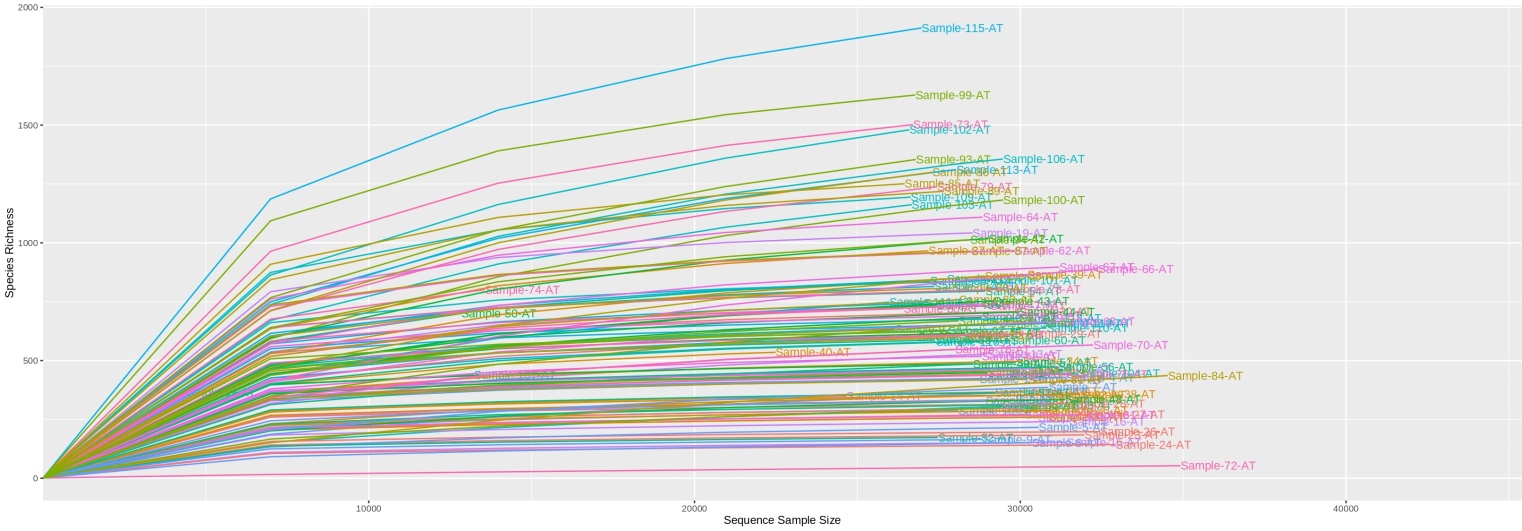


**Supplementary Figure 1.** Rarefaction curves showing species richness coverage generated before any filtration for each milk sample.
